# Realization of user‐friendly bioanalytical tools to quantify and monitor critical components in bio‐industrial processes through conceptual design

**DOI:** 10.1002/elsc.202100116

**Published:** 2021-11-29

**Authors:** Carl‐Fredrik Mandenius

**Affiliations:** ^1^ Unit of Biotechnology Biophysics and Bioengineering IFM Linköping University Linköping Sweden

**Keywords:** bioanalytics, conceptual design, critical process parameters, critical quality attributes, process analytical technology (PAT)

## Abstract

This minireview suggests a conceptual and user‐oriented approach for the design of process monitoring systems in bioprocessing. Advancement of process analytical techniques for quantification of critical analytes can take advantage of basic conceptual process design to support reasoning, reconsidering and ranking solutions. Issues on analysis in complex bio‐industrial media, sensitivity and selectivity are highlighted from users’ perspectives. Meeting challenging analytical demands for understanding the critical interplay between the emerging bioprocesses, their biomolecular complexity and the needs for user‐friendly analytical tools are discussed. By that, a thorough design approach is suggested based on a holistic design thinking in the quest for better analytical opportunities to solve established and emerging analytical needs.

**Abbreviations**
AEnv: active environment
DO: dissolved oxygen
EMA: European Medicinal Agency
FDA: United States Food and Drug Administration
ICH: International Council of Harmonisation
LOD: limit of detection
P&ID: piping and instrumentation diagrams
PAT: process analytical technology
QC: quality control
TrP: transformation process

## 1 THE DESIGNER PERSPECTIVE ON BIOPROCESS ANALYTICS

To acquire relevant information useful for achieving product quality from industrial biotechnology production systems is still a challenge to engineering design although the fundamentals are well known since long [1]. This need is distinctly expressed and motivated by organizations as the International Council of Harmonisation (ICH) [2] and regulatory authorities as FDA [3, 4] and European Medicinal Agency (EMA) in demanding objectives but without providing specific analytical solutions. Although these guidance and recommendations concern mainly pharmaceutical production the basic ideas behind are at large applicable to all biological products such as food, industrial enzymes, or commodity biochemicals [5, 6].

**FIGURE 1:**
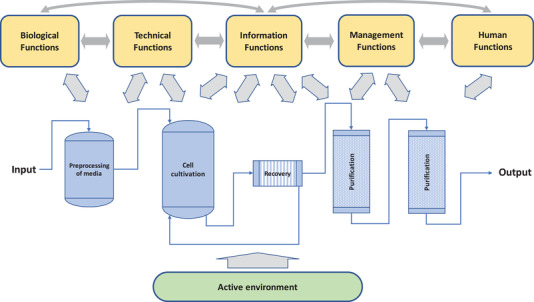
Diagram depicting the operational steps of a bioprocess (here, exemplified by a cell culture process) and the five functional systems influencing its transformation (the biological functions of the cells and the culture media, the bioprocess equipment, the information systems for monitoring, the management procedures for controlling the transformation technically or regulatory, and the humans carrying out the process in the plant). Included is also the active environment that may unanticipatedly influence the process steps or the functions responsible for the transformation

Why does it seem more of a concern to monitor a biotechnology production system than other production systems? The conventional and mostly applied engineering approach in designing processes adheres to view the bioprocess in “piping and instrumentation diagrams (P&ID)” describing the configuration of necessary unit operations for transforming raw materials to a final product [7, 8]. Seldom mentioned in conventional “flow & pipe” mappings are other functions and systems vital for a successful design of a new process. As the Figure 1 shows, such functional aspects of utmost importance for the operativity and transformation in a bioprocess, are the biological, technical, informational, management, and human functions [9]. These are all as critical for succeeding in the design and are all directly or indirectly interrelated with the bioprocess shown in the P&ID. Importantly for the design, all of the functional systems interact in between each other [10]. This in betweenness forms an interactive network often overlooked during the early‐stage process design.

**FIGURE 2:**
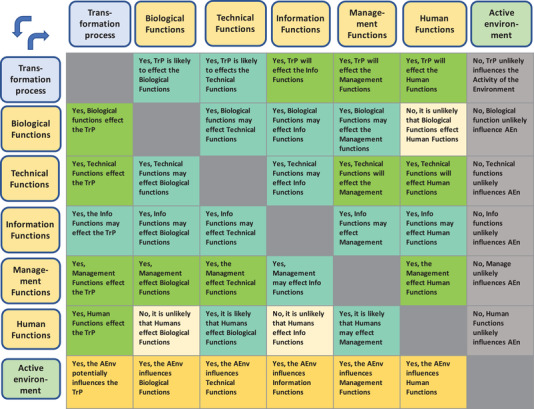
The functions required for the conversion/transformation process (TrP) all interact with it or in between. Also, the undescribed or unknown surrounding environment (AEnv) may actively influence both functions and process. Active means here that it exerts any kind of effect of the functions or process. Note the bend arrows that indicate the direction of the effects (either one function exerts effect on another or the opposite)

To understand the criticality of this interactivity throughout the whole process is of prime concern as indicated with grey bidirectional arrows in Figure 1, and further detailed in the matrix representation in Figure 2. The matrix shows the multiplicity of interactions that are possible but also evaluates their potential influence on the design. For the designer, the bidirectionality is here crucial. Analytical methodologies for acquiring information from the transformation process and from other functional systems involved must bear in mind that these may influence the information itself. Reality is, of course, more complex than the figure implies. Systems responsible for acquiring information in a process involve at least one sub‐level. Still, understanding the interactivity is key to conceiving a successful design of a process information system [11].

Thus, the functionality of the information systems becomes pivotal for both designing and operating the entire bioprocess. Importantly, information in the conceptual chart (Figure 1) includes not only analysis of analytes or components in the process but also their quality, activity, or other derived properties of high value for carrying out and operating the transformation process and the involved functions and subsystems.

This review highlights how the potential and capacity of the information system should cope with the process design issues from the perspective of the users’ actual needs. This becomes very evident in this conceptual approach of process analysis, intending to encompass a broad set of aspects on how to conceive configurations of analytical functions. Thus, to start the bioprocess design by analyzing the functional premises and priorities when configuring the information systems to ensures a more robust structure of process analytics.

## 2 DESIGN OF ANALYTICS THAT SOLVES THE USERS’ NEEDS

Conceiving the design and selection of the information methodology must comply with essential parts of the users´ requirements either it is the biological or technical subsystems driving the process, or the societal and legal regulatory expectations of quality and safety of the product, or the humans operating the bioprocess [11, 12]. This will be highly dependent on the analytical performance with respect to response time, sensitivity, and selectivity of the methodology to meet these specific needs. But also, the design must result in a profound robustness of measurement, signal stability, and cost‐efficiency. Importantly, the efficiency aspects may be extended to include analysis by multiple analytes (i.e. to acquire information about several analytes in the same sample, e.g. through analysis of spectra, 2D electrophoresis mapping, polymerase chain reaction methods or DNA/RNA microarrays). No doubt, many of these aspects of analytical performance and capacity have gone through significant developments in recent decades.

**PRACTICAL APPLICATION**

Successful practice of the guidelines for the design of analytical systems as outlined in this review article may lead to improved functionality and robustness of monitoring and control of bioprocesses. It may also inspire to further develop the analytical technology in directions related to the needs in biomanufacturing practice.

Of imminent significance from the user‐perspective is that the analytical methodologies can inform about the product's quality and safety profiles and how various process functions associated with these profiles are interdependent across the process. The interactivity matrix in Figure 2 identifies these in a structural way. Criteria of observability (response time, sensitivity, variability) as further discussed in Section 3, are naturally decisive if a methodology at all can be considered. But critical from a plant's productivity perspective is also if the methodology can deliver holistic overall information beyond specificity and selectivity.

Table 1 exemplifies the diversity of these aspects for three kinds of information needs, their purposes, and with typical target metrics. Of course, every process and product have unique critical values and metrics. The categories of needs can easily be associated with the objectives mentioned in the cited guidance documents [2, 3, 4] but can as well be identified in the diagrams in Figures 1 and 2.

**TABLE 1:** The industrial needs and targets (typical examples)

Kind of information needs	Specific need (examples)	Target metrics (examples)
Related to target product/quality‐related	• Quantitation of target and level of biomolecular impurities	Percentage (ppm) Structural patterns
	• Verification of target integrity/structure	
Related to plant capacity and production economy	• Bioactivity of cells or biocatalysts to maximize production volumes	Cell specific rates (mass/cell/time) (pH, temp., D.O.) Yield factors
	• Physiochemical conditions for biocatalysts	Target product per plant unit volume
	• Separation in downstream process	
	• Specific efficiency of unit operations	
	• Inhibitive side‐products	
Related to sustainability and environment	• Stability of the production in relation to demands from authorities	Variability (%) at repeated runs
	• Durability of process equipment	Time period
	• Toxic side‐products and leachables from process	Percentage in product fluids and units

Thus, parts of the information concern the biological functions and their variability, other parts the technical functions and their performances, and other parts information, especially information that should be controlled automatically or by operator decisions to ascertain low variability and safe product release.

From the user‐perspective timely information related to critical conditions of a biological product and its biological production system become a priority; information that reveal if:

- The product has the right biomolecular structure (i.e. product integrity)
- Final product state has any toxic component contaminated the production system (i.e. safety for technicians and consumers)
- Final product is freed from harmful components (i.e. side‐effects)
- Stable production levels can be repeated with remained quality (i.e. product release and production economy)
- Enough product is produced to be able to sell to the present market price (business economy)

Without such information production is severely at risk. This requires sufficient sensitivity, selectivity and reproductivity of the measurements. These issues are of greatest concern for every company manufacturing biologics.

**FIGURE 3:**
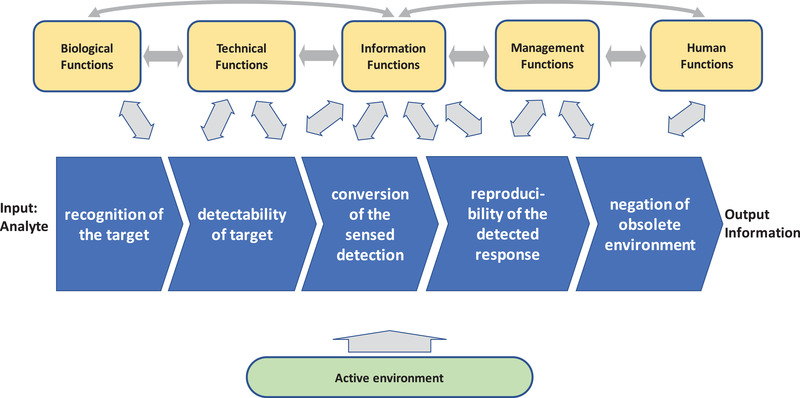
The path for the information system from sampling analytes to reading information through data processing. Each step is crucial for generation of the critical information. The shown path is also a transformation process, alike the bioprocess of Figure 1. Thus, similar functional systems play a vital role in the design of an analytical device, to transform the analyte into reliable and useful information

The design of analytics for process monitoring typical follows the path shown in Figure 3 which principally has the same conceptual structure as Figure 1, and as previously been applied for design of bioanalytical instruments [12] and microfluidics [13].

The transformation stages in the path are the acquisition or capture of the target molecule by a recognition function, often by biomolecular recognition using antibodies, enzymes, or other complementary molecules of biological origin or by molecules able to mimic the complementarity. The recognition event is observed from physiochemical signals transduced by for optical, electronic or heat sensitive sensors. The sensed detection is then converted to useful information, which could be simply concentrations or other parameters of relevance such as growth and production rates. Reproducibility is created by controlled conditions and calibrations. Finally, actions must be taken to compensate for background noise or drift from interfering molecules and components before delivering the information to the user. Often, these actions are compromised by effects caused by the external environment, such as unanticipated interferants in the sample, or variations in the ambient. Examples of the design of analytical instruments where the design teams have been composed of both practitioners of the methodology and design engineers with strong theoretical background enhances successful product development. Examples are the blood glucose biosensor for diabetic care and commercial surface plasmon spectroscopy instruments which both are results of joint design teams.

Conceiving, designing, and implementing a new analytical methodology, easily tends to result in a technology‐driven solution. It is therefore important to drive the design towards user aspects. A functional design approach as in Figure 3 is one step in this direction. This promotes a user‐driven design, while still having technology‐related solutions close in mind. The users have the significant advantage of seeing pros and cons of a design in the intended applications which is not always the case for many skillful inventors. By that, important commercialization aspects of analytical instrumentation are favored.

When developing bioanalytical methodologies, the understanding of biological system is more demanding because of the structural diversity and instability of biomolecular analytes. This must be related to the users’ needs for accomplishing the design solution. Therefore, high priority should be on the biological systems’ ability to:

- Achieve analytical technology with acceptable precision and repeatability according to quality and release criteria.
- Reduce time and effort in operating the analytics (calibrations, maintenance, changing buffers, components, etc.). This may rule out many well‐established analytical techniques but also promising single‐use sensors.
- Respond fast or just‐in‐time, but still with preference to fastest alternatives.
- Reduce burden of additional costs, mainly personnel and other logistics.
- Generate information of value for decision‐making, either directly or by converting data into intelligence of value for approval of quality, enhancing productivity or for process control.

For this to happen, the transformative path in Figure 3 requires a very good complementarity to the analytical components, a high transmissivity of the sensed detection, an inherent specificity in the sensing mechanism, and a resistivity to decay of the sensing ability with time. Moreover, it will be an integral part of the information system shown in Figures 1 and 2 and in between the biological functions but as well as to other functions.

Industrial experiences in implementing PAT [6] have so far revealed reluctance in using new analytical instruments. Reasons could be many, such as limited capacity in meeting the criteria outlined above with accompanying difficulties of being accepted either by the manufacturers’ own personnel. Also, demands from surveillant authorities and market price pressure may be explanations.

Analytical technology successful in meeting all aspects of requirements will however enhance competitiveness, production economy and by that customer satisfaction. Considering that, the conceptual design approach discussed here facilitates in ending up in a design with required information functions.

## 3 PROGRESS OF ANALYTICAL TECHNOLOGY BY USER‐DRIVEN DESIGN

Much attention has been devoted to process monitoring technology over the past decades to design analytics that fulfil the expectations from industry and regulators. This has resulted in analytical designs better in performance in deconvoluting critical process parameters. Table 2 provides an overview of connectivity of the most critical requirements of the bioprocess functions with the strengths of the methodologies and the origin of their uniqueness. These cues direct the selection for making appropriate choices of methodologies for informative purposes. Notably, all of this analytical information is derived from the biological functions in Figures 1 and 3.

**TABLE 2:** Pros and cons with available analytical methodologies

Analytical capacities required in bioprocessing	Methodologies with eminent capacity to respond	Cause of uniqueness (of capacity)
Ability to inform just‐in‐time on biological activity	Spectrometric methods Microscopic methods Calorimetric methods	Fast transformation of light or heat transmission
To detect very low quantities of biological and chemical impurities	Immunologically based methods Biological recognition combined with amplification steps	Sensitive biorecognition between analyte and analytical ligand
To distinguish between structurally very similar molecules in complex media	All methods using Immunological or other biological recognitions such as aptamers, ligands etc.	Unique combinations of biological or non‐biological mimicking structures
To achieve reliable and repeatable information	Methods where the recognition event is stable and protected from degradation or where calibration models are sufficiently reliable	Biostructure is not vulnerable to changes during the time of analysis or use
To deliver information cost‐efficiently in time	Disposable devices with sensor elements Spectrometric methods	Mass fabrication of device, minute consumption of recognition elements

Most of the analytes concerned in Table 2 are easy to analyze by analytical methodologies off‐line in a QC laboratory. But few of these methods can manage with the requirements in response time, sensitivity, stability in crude media, and cost‐efficient operation, at the same time in‐line or near the process.

**FIGURE 4:**
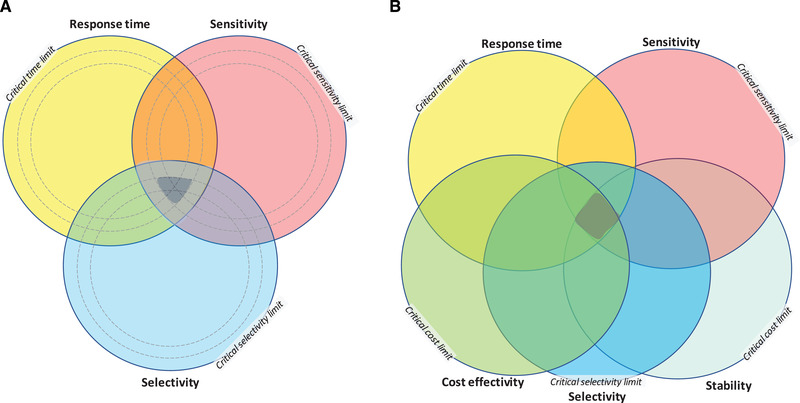
Performance metrics of analytical devices with critical limits as borders. (A) Three critical performances: response time, sensitivity, and selectivity, with three levels of limits and the intersections of these (grey). (B) Extended with two additional metrics, cost‐effectivity and stability, and intersections of all five at critical limit (grey)

To further highlight the requirements in Table 2 they can be translated into maps showing synergies of analytical performances and its critical limit (Figure 4). The actual usefulness of a methodology is indicated by its position (by citation) in the circular areas and their intersections of the maps. Limits in the figure depend on analyte and process. As the arrows and positions of the citations [14, 15, 16, 17, 18, 19, 20, 21, 22, 23, 24, 25, 26, 27, 28, 29, 30, 31, 32, 33, 34, 35, 36, 37, 38, 39, 40, 41, 42, 43, 44, 45, 46, 47, 48, 49, 50, 51, 52] illuminate, many of the methodologies have undergone gradual improvements in performance. These achievements have moved their placement of the circular diagrams towards the intersected areas. But as also apparent in the maps, improvements are desired in several aspects:

### 3.1 Response time

Although just‐in‐time is often a requested capacity in process monitoring it really means the information could await until action based on the information is to take place. Importance of response time is therefore often overvalued. Still, the fastest methodology is preferred if other criteria are attained. Thus, all spectroscopic and microscopic methods are favored due to their responsiveness with the speed of light (near‐infrared spectroscopy [14, 15, 16], multiway fluorescence spectroscopies [17, 18, 19] and Raman spectroscopy [20, 21, 22]). This is also the case with methodologies based on thermal transmission such as calorimetric methods [23, 24] when the conductivity of the used media permits. The strength of these methods is the simplicity of transduction of the signal, compared to methods with biorecognition or other reaction schemes (through antibodies, enzymes, samples treatments, etc.) [14, 15, 16, 17, 18, 19, 20, 21, 22, 23, 24, 25, 26, 27, 28, 29]. Another advantage of spectroscopic techniques is that they can scan wide range of wavelengths and in combination with spectral analysis deconvolute the pattern in models allowing simultaneous quantitation of analytes. This is seldom done with other techniques. However, establishing good model requires time‐consuming acquisition of spectra of components but now computational power has shorted this time. Using in‐line setups with fiber optics real‐time analysis can be done. By that, response time remains short, resolution is much improved although sensitivity and robustness are still rather low.

Microscopy techniques combined with digital image processing are other methodologies favored by speed of light. For long microscopic inspection was tedious work requiring sample preparation but also deep knowledge about microbial structures. Imaging processing can transfer those skills with pattern recognition and machine learning software. Recent ascents in automation of micro‐optical setups have made in situ observation in process fluids possible after sterilizing of the sensitive optic probes inside the process vessels [25, 26, 27]. Developments are considerably but the trade‐off between value of information and cost efficiency of acquiring and using the equipment is high.

With holographic imaging technique the optical advantages remain including capacity of providing real‐time information of cell samples [28, 29]. The technique is still in its infancy despite recent successful commercialization.

Calorimetric methods are favored by rapid signal transduction but only in media able to efficiently conduct heat. However, calorimetry can utilize thermal sensors in heat exchangers in the bioprocess. A bioreactor's cooling and heating system can become an integral part of the measurement system and with heat balancing cell growth and concentration can be estimated. But proper model calibration requires digital solutions [26, 27].

**FIGURE 5:**
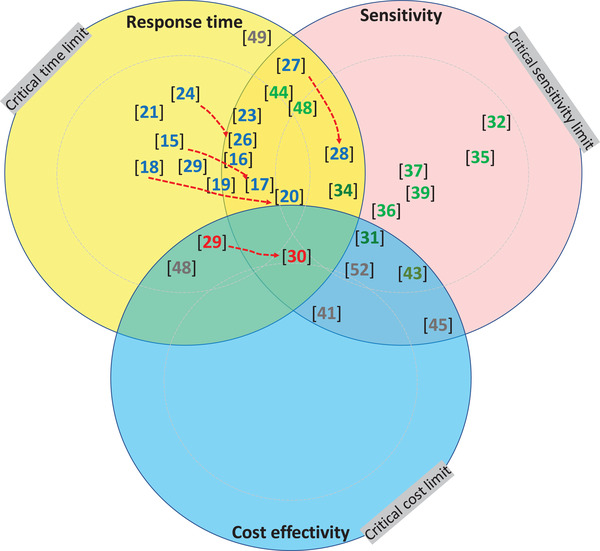
Analytical performance with critical limits for response time, sensitivity, selectivity, and cost effectivity. Selected methodologies are cited in the diagram: near‐infrared‐ [15, 16, 17], 2D fluorescence‐ [18, 19, 20], and Raman spectroscopies [21, 22, 23], in‐situ microscopy [24, 25, 26], holography [27, 28], calorimetry [29, 30], enzyme electrodes [31], enzyme thermistors [32, 33, 34], localized surface plasmon resonance [35], immuno‐capacitive sensors [36, 37, 38, 39], screen‐printed electrodes [40], aptamers [45], molecular imprinted polymers [47], electronic noses [48] and tongues [49], and lateral flow sensors [52]. Optical and spectroscopic methods are labelled blue, calorimetric red, methods based on biorecognition green, other methods with grey. Dashed red arrows show advancement of methodologies over time

These rapidly responding information systems are attractive for process monitoring and abide intentions with many of the PAT initiative's objectives [6]. However, as apparent in Figure 5, limits for sensitivity and selectivity are not reached sufficiently well.

### 3.2 Selectivity

As mentioned above, sufficient selectivity of the methodologies in complex process media is pivotal for their usefulness. Here, methods exploiting biorecognition exhibit superior selectivity for molecule with minute structural differences. Unfortunately, many of the biorecognition methods suffer from instability, due to decay or shift of specificity, when recognition sites deteriorate. To negate this, various preparation steps such as pre‐purification of samples and addition of stabilizing reagents are necessary. Despite this, many of these methods have a place in bioprocess monitoring, at least when applied at‐line or offline in QC labs. Methods based on immunoanalytical‐ and nucleic acid hybridization procedures show outstanding selectivity (e.g. enzyme‐linked immunosorbent assays, surface plasmon resonance spectroscopy, polymerase chain reaction, and DNA/RNA microarrays) but their complex assay protocols are seldom realizable in‐line. Still, several successful demonstrations are carried out for bioprocess monitoring (e.g. with biosensors based on enzyme electrodes, microgravity sensors, and thermal biosensors) but few are used in industrial practice and if so, only in process R&D. Successful commercially examples could be noted, such as the BioPAT system (Sartorius) using enzyme electrode [30], the enzyme thermistors [31, 32] showing wide versatility in applicability [33], flow injections analysis systems [34] and localized surface plasmon resonance spectroscopy [35]. Most of these have been successful tried in in‐line setups but then with demanding auxiliary automation which probably explain their sparse use at industrial scale.

### 3.3 Sensitivity issue

As already emphasized above, sensitivity of the methodologies is as pivotal for their applicability. Usually the limit‐of‐detection (LOD) defines the sensitivity but must be paired with the precision at the LOD to be a useful measure. Most of occurring impurities need to be detected close to the LOD while product molecule should normally be quantified at much higher titers and preferably simultaneously. This is a profound challenge since product targets and impurities are to at large extent structurally close. Again, biorecognition methods are favored owing to both selectivity and sensitivity, given efficient signal transduction of the recognition event is available. This can be accomplished with many biosensors using a variety of biorecognition elements such as antibodies or enzymes, as quoted [31, 32, 33, 34, 35], and where amplification methods can be employed for lowering the LOD if so needed. Promising attractive sensor solutions for very low concentrations of process impurities are immuno‐capacitance sensors with antibodies [36, 37] and new developments of thermistor technique [33]. However, long response time for delivering critical information may become a severe obstacle; procedures required for bringing the analyte into a state that can be sensitively and specifically measured retards the analysis. If automation of that procedure can be realized reliably and cost‐effectively, disposable sensors or low‐cost flow systems can be attractive solutions as shown for the VERSAFLO technique [34], localized surface plasmon resonance [35], and screen‐printed sensors [38]. Another interesting alternative is lateral flow techniques due its combined high sensitivity and low fabrication cost [52]. The positioning of these methodologies in the sensitivity circle in Figure 5, mostly correlated with selectivity, is noteworthy although response limits are not often reached.

### 3.4 Stability

Analytical stability and reproducibility are usually attained by recalibration of the instrumental setup. Very few methods exhibit real long‐term stability. Most vulnerable to destabilization are analytics using biological recognition because of declining activity of recognizing biomolecules, loss of active structure, fouling or irreversible blocking of sites or thermal denaturation. Decisive is what actual stability is needed for making the information useful. How long stability is required? Is calibration realistic in manufacturing? How often must a method undergo revalidation? For this, some methodologies are better choices: e.g. calorimetry which response is insensitive to fouling; spectroscopic methods, independent of recognition elements and able of using spectral models, demanding to establish, but easy to tune; commercial disposable sensors using pre‐calibrated recognition element only once. Of particular interest in this context are new recognition molecules such as imprinted ligand molecules [47] and synthesized aptamers that mimic biological structures with higher long‐term stability than the mimicked biorecognition sites [43, 45].

### 3.5 Cost‐effective operation

The investment cost of an analytical instrumentation may be a hurdle for its implementation but to that must be added costs of management, operator time, maintenance, spare‐parts and consumables, data interpretation, trouble‐shooting and other disposables, all related to cost‐effectiveness. Naturally, the users want to avoid these expenses. Cost‐efficiency for a methodology should primarily be understood as how it unburden of production cost for the plant and not as price per assay. Consequently, reliable disposable or single‐use devices are here advantageous, they have a low one‐time permanent unit price, low cost for establishing a procedure (SOP), low operator training, and low validation cost [e.g. 40, 42.]. Spectroscopic methods on the other hand require extensive investments, method development, training, competence of personnel involved and thereby easily become both a high cost and logistic burden. This influences considerably prerequisites for the design of analytics. Cost‐effectivity is also indicted in Figure 5.

### 3.6 Validity

For all process analytics validation are demanding and strictly prescribed by authorities in pharma and partly also be in other life science industries. Questions on how generally applicable the methodology is, how much the vendor must adapt devices and protocols and if the validity is sufficiently durable over time under realistic operational conditions, should be satisfactorily investigated. Even if these issues seem like one‐time issues, they must be convincingly overcome before integrating the analytics into the plant and its operation. The methodologies mentioned here are obviously very different in terms of validation. The validity is of course also dependent on the actual process making it difficult to foresee how demanding validation may be. The reliability of the validation in the bioprocess becomes of high priority and a decisive ranking factor in the design.

### 3.7 Simultaneous measurement of analytes

In accordance with what said above about cost efficiency, methodologies able to deliver analytical information for more than one analyte have a strong advantage. Well‐known such methods are the polymerase chain reaction methods, DNA‐RNA microarray, 2D‐electrophoresis, and spectral methods where each spectrum contains information about several analytes. However, these methods suffer from being labor‐demanding and therefore disfavored as process analytics tools. Still, many single‐use devices, have potential to cover several analytes using arrays of stable recognition elements, e.g. aptamers [43, 45]. These methodologies are from user perspectives attractive but must first pass demanding validation procedures. Techniques with miniaturized electronic multi‐sensors (e.g. electronic noses and electronic tongues) seem usefulness especially for in place measurement with online computation of their response pattern. Close to this is to compute response patterns with engineering equations and models for cellular kinetic behavior to reveal multivariate information (e.g. growth rate, cellular respiration, inhibitions), sometimes referred to as soft sensors.

Figure 4 illuminates analytical methodologies’ dependency on combinations of critical performance measures. In Figure 4A three important performance criteria are considered: sensitivity, selectivity, and response time at three levels of limits. Intersections of these show smaller intersecting areas when limits are tightened. In Figure 4B, the cost effectivity and stability performance are added. As illustrated, the intersection area with five shrinks even more. The critical limits in the diagrams are here arbitral; their levels alter pros and cons for the methods. Each bioprocess has specific limits and critical parameters, e.g. for impurities. And importantly, levels will vary along the process train of unit operations. Here, the regulators’ demand, but also the quality policy of the manufacturing company, and the plant influence critical levels. Nevertheless, the outcome of the graphics of the diagrams is obvious, the performance of analytics should be driven further in directions towards the users’ needs.

## 4 CONCLUDING REMARKS

To summarize, the design perspectives outlined in this minireview provide alternative approaches in the quest for better bioprocess analytical technology for the monitoring and control of bioproduction. Major conclusions are that:

- Several analytical methodologies can fulfil critical information needs such as response time, parallel analyses, and specific measurement but not within the scope of the same methodology and setup. Especially measurements in complex biological environments with multitudes of interfering molecules in wide ranges of concentrations and minute structural differences are demanding.
- Sensitivity of fast‐responding methodologies suffice for a variety of non‐critical quality attribute, while most critical analytes, or critical quality attributes, including impurities and toxic components, have concentrations that fall below limits of detection to allow real‐time measurements. This leaves out many convenient and appealing methodologies unable to trade‐off for example speed of detection for sensitivity. Proteins especially, with their structural diversity, require very specific recognition methods where a rapid response time must be sacrificed.
- Attractive disposable sensors lack cost‐efficiency if they become too labor‐intensive, whereas under favorable conditions, such obstacles can be circumvented.
- Methodologies that measure critical quality attributes fast enough, preferably in parallel, sensitively, and stably enough are so far not realized. If the information functions of a bioprocess are designed by paying more attention to the overall information needs for the production with realistic user priorities, more realistic analytical configurations can be developed. This is especially true from the perspective of developers of commercial analytics.
- Progress of general analytical science, methodologies, and techniques for bioprocessing applications, would benefit from driving continuing development both by priority to user needs and emerging technology.

## CONFLICT OF INTEREST

The author declares no conflict of interest.

## Data Availability

The data that support the findings of this review are available from the corresponding author upon reasonable request.
